# Gene therapy to cure HIV? Prospects and realities

**DOI:** 10.1186/1742-4690-9-S1-I3

**Published:** 2012-05-25

**Authors:** Gero Hütter

**Affiliations:** 1 Heidelberg University, Württemberg, Germany

## Introduction

HIV-1 can persist in a latent form in resting memory CD4+ cells and macrophages carrying an integrated copy of the HIV genome. Because of the presence of these stable reservoir cells, eradication by antiretroviral therapy is unlikely and in order to achieve eradication, alternative treatment options are required.

## Materials and methods

Recently, we have described a successful hematopoietic stem cell transplantation in an HIV-1 infected patient transferring donor derived cells with a natural resistance against HIV infection. These haematopoietic stem cells engrafted, proliferated and differentiated into mature myeloid and lymphoid cells.

## Results

At present the patient is more than five years after allogeneic transplantation without requirement of any antiretroviral treatment. Analyzing peripheral blood cells and different tissue samples including gut, liver, and brain, no viral load or proviral DNA could be detected.

## Conclusions

There is a degree of optimism that gene therapy in combination with SCT can improve HIV-1 treatment. After nearly 30 years of research and progress HIV infection has become a chronic disease in developed countries. However, the evolution of this infectious agent, due to its rapid mutation, is quite unpredictable. Therefore, strategies to eliminate the virus from the body to achieve cure are desirable. Currently, HIV-targeted gene therapy appears to have great potential as an effective strategy that may eliminate or reduce the need for antiretroviral therapy and achieve elimination of the viral reservoir.

